# Supplementary material for: Anapc5 and Anapc7 as genetic modifiers of KIF18A function in fertility and mitotic progression
Source: bioRxiv. 2024 Dec 4:2024.12.03.626395. Preprint. [Version 1] doi: 10.1101/2024.12.03.626395 (PMC11642851; doi:10.1101/2024.12.03.626395)
Supplement: 1 [file NIHPP2024.12.03.626395V1-supplement-1.pdf]

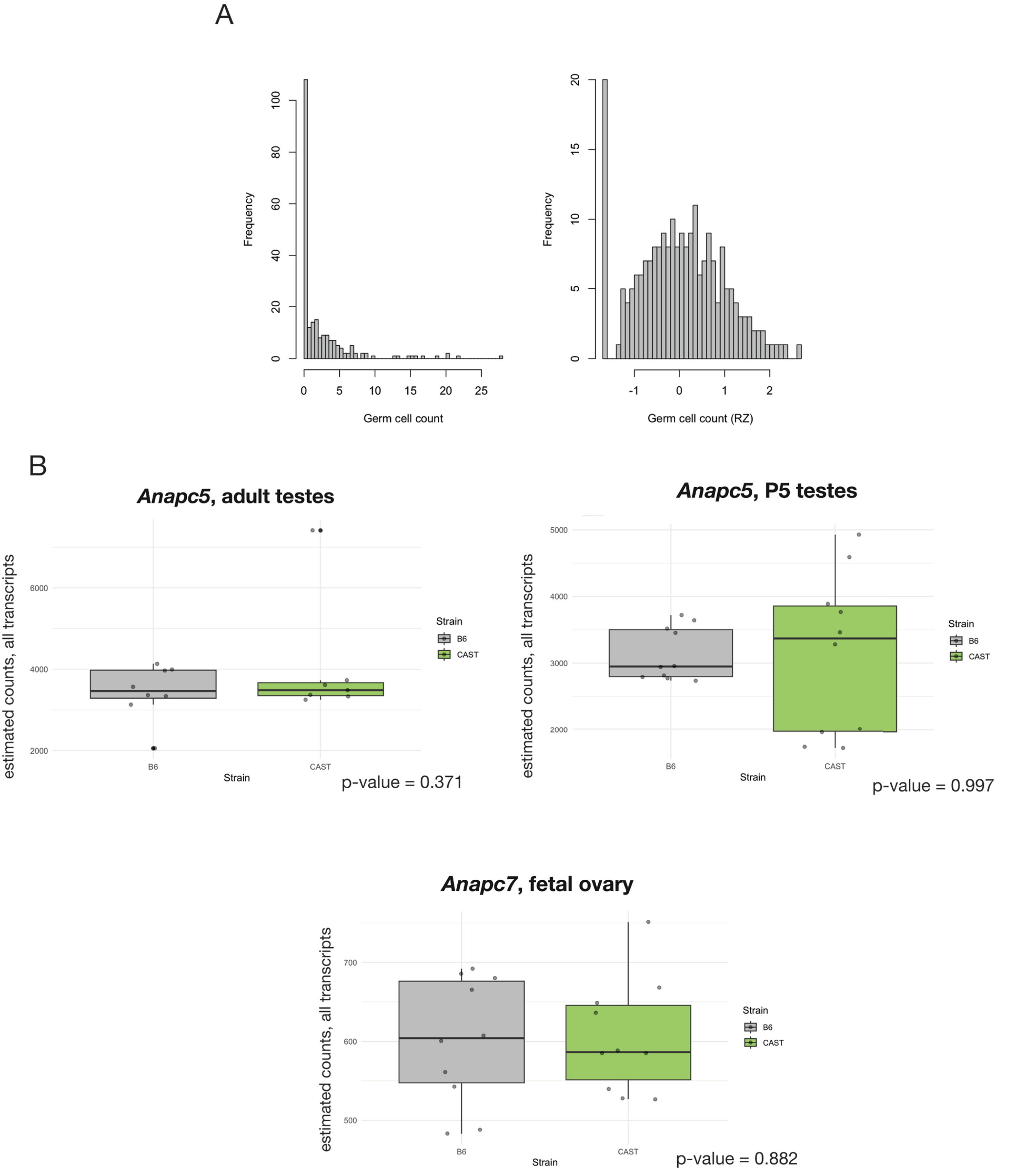

Supplementary Figure 1. Variation of mouse germ cell populations in B6;CAST-*Kif18a*$^{gcd2/gcd2}$ F2, raw data and RankZ transformed (A). Tissues and timepoints where *Anapc5* and *Anapc7* expression differences between B6 and CAST were not significant, n=5 for each tissue type/strain (B).

bioRxiv preprint doi: https://doi.org/10.1101/2024.12.03.626395; this version posted December 4, 2024. The copyright holder for this preprint (which was not certified by peer review) is the author/funder, who has granted bioRxiv a license to display the preprint in perpetuity. It is made available under aCC-BY 4.0 International license.

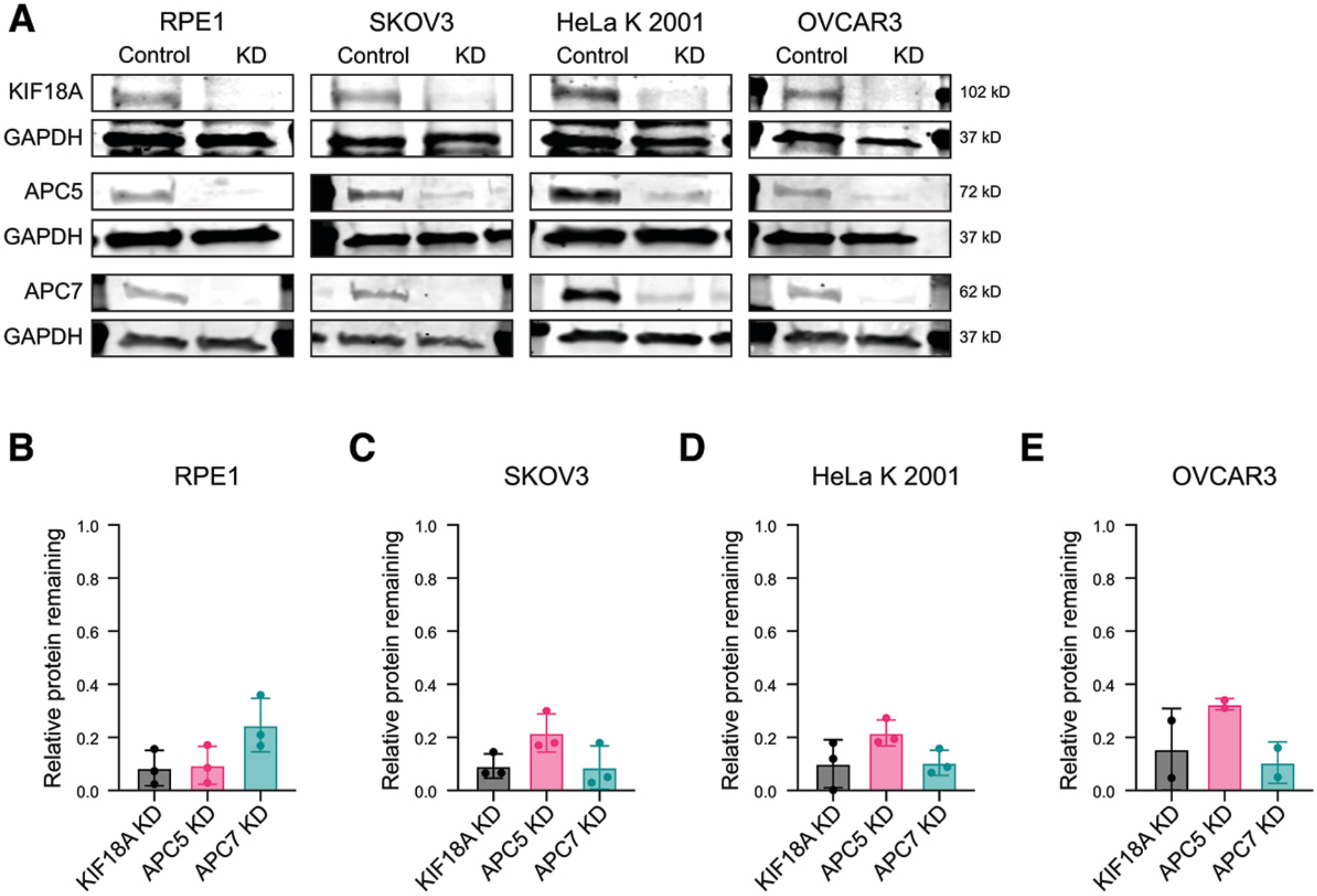

Supplementary Figure 2. Representative Western blots of proteins of interest following knockdown (KD) with siRNA in RPE1, SKOV3, HeLa, and OVCAR3 cells (3 independent experiments for each protein for RPE1, SKOV3, and HeLa cells, 2 independent experiments for each protein for OVCAR3 cells.) (A) **B,C,D,E** Quantification of siRNA KD in RPE1 (B), SKOV3 (C), HeLa (D), and OVCAR3 (E) cells measured via Western blot and normalized to control siRNA condition and GAPDH loading control. n=3 individual 3 independent experiments for each protein for RPE1, SKOV3, and HeLa cells (all conditions), 2 independent experiments for each protein for OVCAR3 cells (all conditions).
